# Retraction Note to: Vitamin A deficiency and sleep disturbances related to autism symptoms in children with autism spectrum disorder: a cross-sectional study

**DOI:** 10.1186/s12887-021-03000-8

**Published:** 2021-12-03

**Authors:** Jing Wen, Ting Yang, Jiang Zhu, Min Guo, Xi Lai, Ting Tang, Li Chen, Jie Chen, Ming Xue, Tingyu Li

**Affiliations:** 1 Children’s Nutrition Research Center, Children’s Hospital of Chongqing Medical University, Chongqing Key Laboratory of Child Nutrition and Health, Chongqing, PR China; 2 Ministry of Education Key Laboratory of Child Development and Disorders, Chongqing, PR China; 3 National Clinical Research Center for Child Health and Disorder, Chongqing, PR China; 4 China International Science and Technology Cooperation Base of Child Development and Critical Disorders, Chongqing, PR China; 5 Department of Neurosciences and Neurology, Rutgers New Jersey Medical School, Newark, NJ USA

**Retraction Note to: BMC Pediatr 21, 299 (2021)**

**https://doi.org/10.1186/s12887-021-02775-0**

The Editor has retracted this article because of concerns with the data presented and ethics approval for this study. After publication, the authors became aware of fundamental errors in the data presented in Tables 2 and 3 that undermined the conclusions drawn in this study and contacted the journal to request a retraction. Additional concerns have been raised about the Ethics Approvals for the study since the protocol was updated, but the authors were unable to provide evidence that these changes were approved by all relevant Institutional Review Boards.

Authors Ting Yang, Xi Lai, Li Chen, Jie Chen, Ming Xue and Tingyu Li agree to this retraction. Authors Jing Wen, Jiang Zhu, Min Guo and Ting Tang have not responded to any correspondence from the editor or publisher about this retraction.

